# Lung Inflammation Is Associated with Preeclampsia Development in the Rat

**DOI:** 10.3390/cells11121884

**Published:** 2022-06-10

**Authors:** Katrina Curtis, Derek Clarke, Makayla Hanegan, Brendan Stapley, Ryan Wendt, Nathan Beckett, Cade Litchfield, Kennedy Campbell, Paul Reynolds, Juan Arroyo

**Affiliations:** Lung and Placenta Laboratory, Department of Cell Biology and Physiology, Brigham Young University, Provo, UT 84602, USA; klbreit7@gmail.com (K.C.); clarke1416@gmail.com (D.C.); mkay.hanegan@icloud.com (M.H.); brendan.stapley@yahoo.com (B.S.); ryan.wendt00@gmail.com (R.W.); nathancbeckett@gmail.com (N.B.); cadelichfield@gmail.com (C.L.); 3ksoup@gmail.com (K.C.); paul_reynolds@byu.edu (P.R.)

**Keywords:** preeclampsia, lung, inflammation, Gas6, AXL

## Abstract

Preeclampsia (PE) is an obstetric complication associated with significant health implications for the fetus and mother. Studies have shown a correlation between lung disease development and PE. Gas6 protein is expressed in the lung and placenta, and binds to the AXL Tyrosine kinase receptor. Recently, our laboratory utilized Gas6 to induce preeclamptic-like conditions in rats. Our objective was to determine the role of Gas6/AXL signaling in the maternal lung during PE development. Briefly, pregnant rats were divided into control, Gas6, or Gas6 + R428 (an AXL inhibitor). Immunofluorescence was performed to determine AXL expression. Bronchoalveolar lavage fluid (BALF) was procured for the assessment of inflammatory cell secretion. Western blot was performed to detect signaling molecules and ELISA determined inflammatory cytokines. We observed increased proteinuria and increased blood pressure in Gas6-treated animals. AXL was increased in the lungs of the treated animals and BALF fluid revealed elevated total protein abundance in Gas6 animals. Extracellular-signal regulated kinase (ERK) and protein kinase B (AKT) signaling in the lung appeared to be mediated by Gas6 as well as the secretion of inflammatory cytokines. We conclude that Gas6 signaling is capable of inducing PE and that this is associated with increased lung inflammation.

## 1. Introduction

Proper placenta function is important for a successful pregnancy. Several obstetrics complications are associated with placental dysfunction such as those observed during PREECLAMPSIA (PE). PE is an obstetric complication characterized by high blood pressure after the 20th week of pregnancy; 140 mm Hg (systolic) or 90 mm Hg (diastolic) and increased protein in the urine (≥300 mg in 24 h) [1]. This obstetric complication accounts for up to 20% of preterm births, increased intrauterine fetal demise (IUFD), and the development of adult hypertension, heart disease, stroke, and diabetes [2,3,4,5]. Recent research has shown that there is an increase in Gas6 protein in the serum of preeclamptic patients, highlighting Gas6 as a perpetuation factor in PE severity [6]. Gas6 is a vitamin K-dependent protein expressed in the lung, heart, kidney, and intestine, and is detectible in human plasma [7]. Gas6 serves as a ligand to the Tyro3, AXL, and Mer Tyrosine kinase receptors, having the highest affinity for AXL receptors [7,8]. AXL is a transmembrane receptor tyrosine kinase (RTK), and Gas6/AXL pathways are known to be involved in several diseases including cardiovascular pathologies [9]. Recently our laboratory has developed a rodent Gas6 model of PE [1]. This model showed PE-like characteristics including increased blood pressure, increased proteinuria, increased placental-derived inflammation, decreased trophoblast invasion, and an increase in placental apoptosis (gas6 paper). Inhibition of the Gas6 receptor AXL, reduced these PE-like characteristics observed in this rat model of PE [1]. Together these studies suggest an important role for Gas6 and its receptor AXL in the development of this obstetric complication. Although the AXL receptor is expressed in the placenta, its expression is much higher in the lung. This caused us to question the role of the Gas6/AXL pathway in the lung in this model of PE. This is important as previous reports had shown the development of lung diseases, such as lung edema, and the presence of placental syncytial aggregates in the lungs of mothers during preeclampsia [10,11,12,13,14,15,16,17]. Many of the Gas6/AXL studies have concentrated on lung cancer, but not much is known about other different environments where Gas6 and AXL are affected as those observed during PE. Our objective was to determine Gas6/AXL signaling in the maternal lung in this Gas6-induced model of PE.

## 2. Materials and Methods

### 2.1. Animals and Tissue Preparation

Brigham Young University Animal Care and Use Committee (IACUC) approved this study (Approval number PRE21-0012). Pregnant Holtzman Sprague Dawley (HSD) rats (Weight-matched; ~400 g) were necropsied at 18.5 days of gestation (dGA). At this point, placental, and fetus weights were recorded, and placental and lung tissues collected in liquid nitrogen for protein analysis. Lung tissues were paraformaldehyde (PFA) inflation fixed prior to processing, embedding, and sectioning for immunofluorescence (IF) analysis [18]. Lung and placental tissue samples were stored at −80 °C until used.

### 2.2. Animal Treatments

PE pregnancy was generated as previously performed in our lab [1]. PE was observed by the i.p. administration of recombinant Gas6 protein (R&D, Minneapolis, MN, USA) to pregnant rats. The dose of Gas6 was in accordance with several other research endeavors that pursued a Gas6 dose-response and disease modeling in rats, which we have previously reported [1]. Briefly, starting at day 7.5 dGA, pregnant rats were injected with Gas6 (at a concentration of 4 μg/kg of body weight) for 11 days (to day 17.5 dGA; Gas6 animal group; n = 10). A Control group of pair-fed animals was administered saline injections (n = 10). For AXL inhibition studies, Gas6-treated pregnant animals (n = 10) were treated with a daily i.p. injection of R428 (and 75 mg/Kg; APExBIO, Houston, TX, USA) for 4 days (Gas6 + R428 group, treatment starting at day 13.5 dGA to day 17.5 dGA).

### 2.3. Blood Pressure

A CODA monitor system (CODA tail-cuff blood pressure system; Kent Scientific Corporation; Torrington, CT, USA) was used to measure blood pressure as previously performed in our laboratory [1]. This system includes a heating pad and a fully automated occlusion tail cuff. During blood pressure measurements, animals were restrained for 5 min by a medium-sized clear column crafted by Kent Scientific. Blood pressure measurements were performed daily in the control and treated (Gas6 and Gas6 + R428) animals.

### 2.4. Proteinuria 

Proteinuria levels (n = 10 per group) were determined using a dipstick approach to confirm PE (characterized by detecting proteinuria at the +3 and +4 levels). At the time of necropsy, urine was collected and dipstick color development was evaluated. The urine dipstick test (Siemens Urinalysis Test Uristis^®^ strips; Siemens; Malvern, PA, USA) was performed following the manufacturer’s instructions. Categories included negative, trace, +1 (30 mg/dL), +2 (100 mg/dL), +3 (300 mg/dL), and +4 (≥2000 mg/dL). 

### 2.5. RNA Isolation and Analysis

Isolation and analysis of RNA were performed as previously performed in our lab [1]. Briefly, after isolation, RNA was quantified using a Nanodrop. Bio-Rad iTaq (Universal SYBR^®^ Green One-Step Kit was used to amplify cDNA (Bio-Rad, Hercules, CA, USA). A Bio-Rad Single-Color Real-Time PCR detection system (Bio-Rad Laboratories, Hercules, CA, USA) was used for data analysis. The following primers were synthesized by Invitrogen Life Technologies (Grand Island, NY, USA): AXL (For-CTAC GAG ACG TCA TGG TAG and Rev-GCT CTG ATC TTG TGC AGA TG), and β-actin (For-ACA GGA TGC AGA AGG AGA TTA C and Rev- CAC AGA GTA CTT GCG CTC AGG A).

### 2.6. Immunofluorescence

Paraffin-embedded lung sections (n = 5) were used for Immunofluorescence (IF) studies as previously performed in our laboratory [1]. In summary, after blocking, lung slides were incubated overnight with a mouse primary antibody against AXL. The next day, a secondary donkey anti-mouse Texas Red (TR; Santa Cruz Biotechnology, Santa Cruz, CA, USA) was incubated for an hour. IF detection was performed using a BX6 microscope.

### 2.7. Bronchoalveolar Lavage Fluid (BALF)

BALF was performed in treated and control pregnant rats (n = 10) as outlined previously with slight modifications [19]. Briefly, control and treated animal lungs were inflated and fixed with 4% PFA for lavage to procure BALF. This was specifically harvested through the installation and recovery of seven boluses of PBS with a syringe attached to a catheter for a total of 30 mL/kg. BALF samples were centrifuged for 10 min and supernatants were assayed for total protein using a bicinchoninic acid (BCA) total protein kit (Thermo Scientific). Pelleted cells were counted and stained with a modified Wright–Giemsa stain (Diff-Quik; Baxter, McGaw Park, IL, USA) for a blinded manual differential cell count in which 200 cells were counted per slide to determine the percent of total cells.

### 2.8. Immunoblotting 

RIPA protein lysis buffer (Fisher Scientific, Pittsburg, PA, USA) was used to homogenize lung tissues (n = 6 per group) used for the detection of extracellular signal-regulated kinase (ERK) and protein kinase B (AKT) protein-signaling molecules as previously described in our laboratory [9]. Briefly, lung protein lysates (20 mg) were separated using a Mini-PROTEANV^â^ TGX^TM^ Precast gel (Bio-Rad Laboratories, Hercules, CA, USA) followed by transfer to nitrocellulose membranes. Membranes were incubated overnight with antibodies against phospho ERK and AKT proteins (Cell Signaling, Danvers, MA, USA). Fluorescence tagged secondary antibodies were added for one hour and fluorescence emission was digitally recorded using a C-DiGitV^â^ Blot Scanner (LI-COR, Inc., Lincoln, Nebraska). To confirm loading consistencies, each membrane was stripped and re-probed utilizing an antibody against actin (Cell Signaling, Danvers, MA, USA). Average band densities (of at least twice in triplicate experiments) were normalized to b-actin densities prior to performing statistical tests.

### 2.9. ELISA

BALF collected at the time of necropsy (n = 10) was used to screen inflammatory mediators. Secreted Interleukin 1 alpha (IL-1α), Interleukin 2 (IL-2), and tumor necrosis factor alpha (TNFα) levels were assessed using colorimetric high-throughput fast activated cell-based ELISA assays (RayBiotech; Peachtree Corners, GA, USA) using an Epoch Microplate reader (Biotek Instruments Inc.; Agilent; Santa Clara, CA, USA). For this, equal volumes of lung BALF were assessed in each experimental group of treated and control animals as directed by the manufacturer.

### 2.10. Statistical Analysis

Differences in ER and AKT protein activation, and cytokine protein expression were determined between control and treated (Gas6 and Gas6 + R428) pregnant animals using Mann–Whitney tests. GraphPad Prism 7.0 software (GraphPad; Santa Clara, CA, USA) was used for statistical analysis and significant differences in the data are shown as means ± SE.

## 3. Results

### 3.1. Gas6 Induces PE in Rats

**Figure 1:**
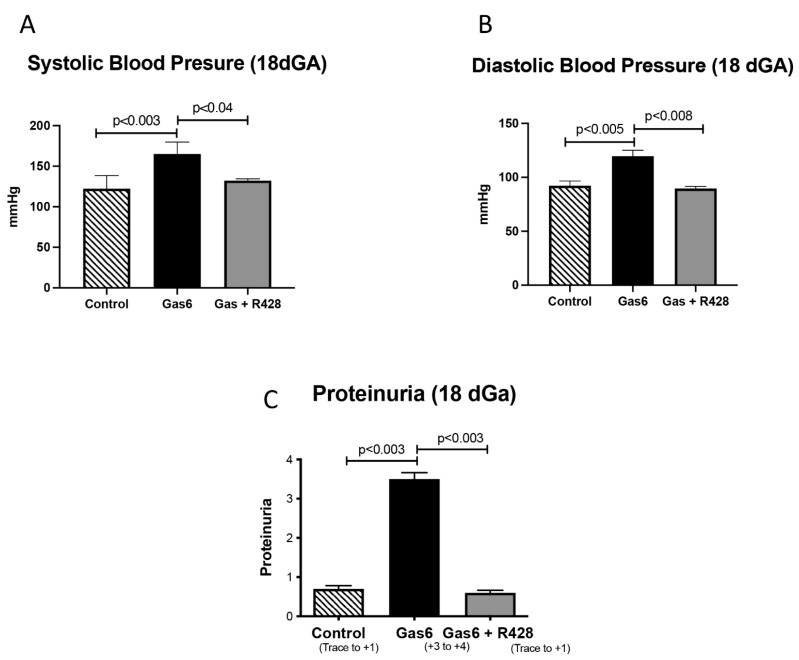
Blood Pressure and proteinuria during Gas6 treatment in the pregnant rat. There was an increase in blood systolic (**A**) and diastolic pressure (**B**) and urine proteinuria (+3 and +4) (**C**) in treated animals as compared to controls (n = 10). Systolic (**A**) and Diastolic (**B**) blood pressures and proteinuria (**C**) returned to basal levels in animals treated with Gas6 and the Axl inhibitor as compared to those treated with Gas6 alone. Representative data are shown with *p* ≤ 0.05.

To induce PE, pregnant rats were treated with Gas6 for 11 days [1]. We observed that Gas6 treatment increased both Systolic and Diastolic blood pressure in pregnant dams at the time of necropsy (Figure 1A,B). This increase in blood pressure was reversed when the AXL receptor was inhibited by treatment with R428 (Figure 1A,B). We next investigated proteinuria in the urine of control and treated animals. There was significantly increased proteinuria (+3 to +4) in Gas6 animals compared to controls, which was reversed to basal levels (trace to +1) when the AXL receptor was inhibited (Figure 1C).

### 3.2. Lung and Gas6 Treatment

**Figure 2:**
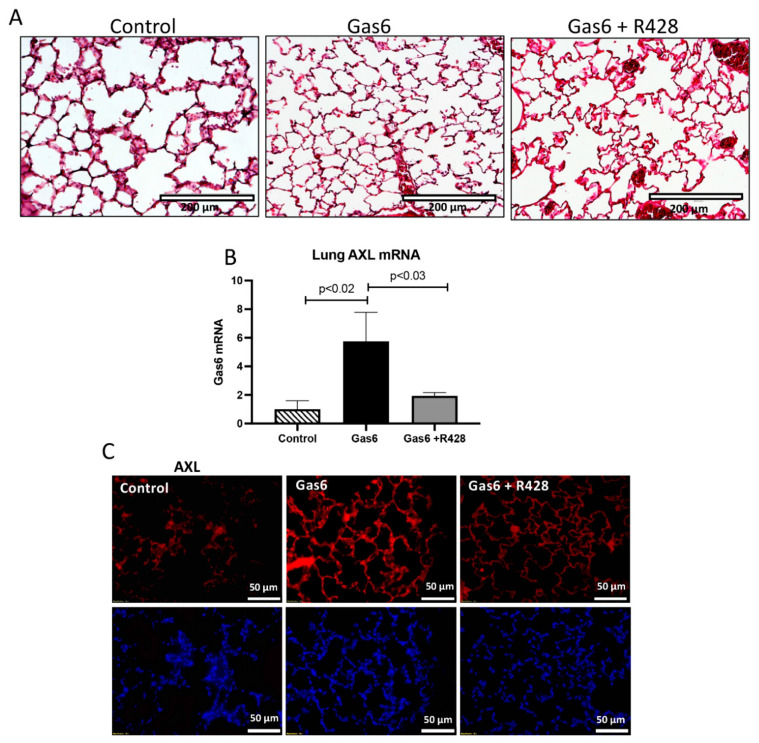
Lung AXL expression during Gas6 treatment in the pregnant rat. Hematoxylin staining was performed for lung structure determination (**A**). There was an increase in AXL mRNA in the treated animals as compared to controls (**B**). This increase was also observed in AXL protein levels in the lung of treated animals as compared to controls (**C**).

**Figure 3:**
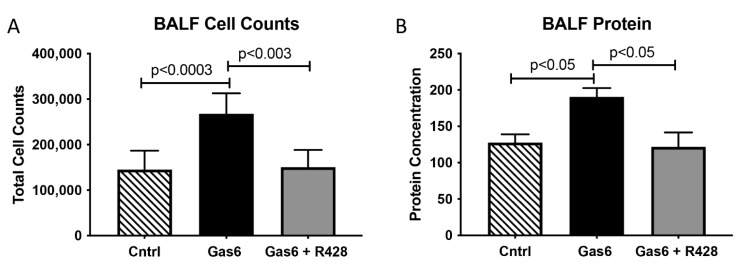
BALF analysis in control and treated animals. Cell count (**A**) and protein (**B**) were increased in the BALF of Gas6-treated animals as compared to controls. Levels were decreased in animals co-treated with R428 and Gas6 (n = 10). Representative data are shown with *p* ≤ 0.05.

We first determined AXL levels in the lungs of treated animals vs. controls. Lung histology is shown in Figure 2A. We observed an increase in AXL mRNA (5.7-fold; *p* < 0.02) in Gas6-treated animals as compared to controls (Figure 2B). This increase in gene activation was reduced with the addition of the AXL inhibitor (Figure 2B). Next, we used immunofluorescence to determine AXL expression patterns in the lungs of control and PE animals. We observed that AXL protein expression was increased in the animals treated with Gas6 as compared with the controls and that the addition of the AXL inhibitor reduced AXL presence in the lungs of these treated animals (Figure 2C). BALF was used to determine lung cell infiltration due to AXL activation by Gas6. We observed that the number of infiltrating cells (leukocytic cells) was increased (1.8-fold; *p* < 0.003) in the lung of the PE animals as compared to controls (Figure 3A). This increase in cell counts was decreased to basal levels when the AXL receptor was inhibited (Figure 3A,B). Similarly, BALF protein quantification showed an increase in protein levels (1.5-fold; *p* < 0.05) during Gas6 treatment that was reduced to basal levels by the addition of the AXL inhibitor.

### 3.3. Lung-Signaling Molecules and Released Cytokines 

**Figure 4:**
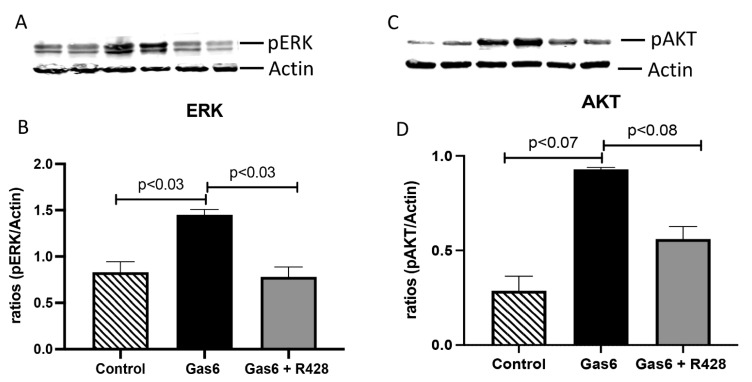
Lung-signaling molecules in control and treated animals. A representative Western blot for ERK and Akt is shown in (**A**,**C**). Lung levels of ERK (**B**), and AKT (**D**) were increased in Gas6-treated animals compared to controls (n = 6 per group). Molecules were deceased in animals co-treated with R428. Representative data are shown with *p* ≤ 0.05.

**Figure 5:**
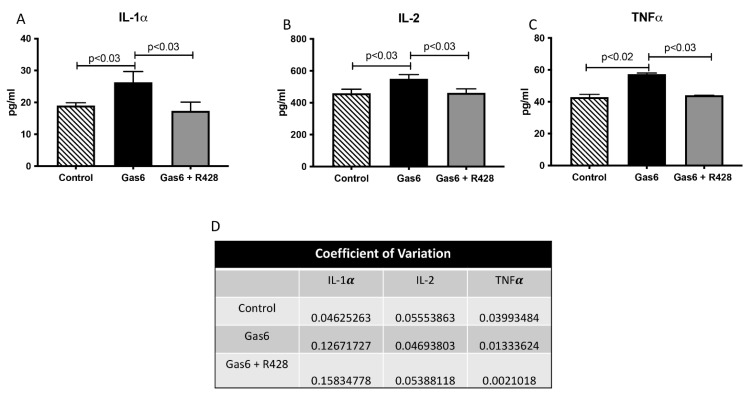
Inflammatory cytokines in the serum of control and treated animals. Serum levels of IL-1𝜶 (**A**), IL-2 (**B**), and TNF𝜶 (**C**) were increased in Gas6-treated animals compared to controls (n = 10). Cytokines were deceased in animals co-treated with R428. Coefficients of variations for the experiments are found in (**D**). Representative data are shown with p≤ 0.05.

We next wanted to determine lung-signaling molecules in this model of PE. Specifically, we studied levels of ERK and AKT kinases that signal transduction and are activated in Gas6/AXL signaling [8,20]. A characteristic Western blot for ERK and AKT is shown in Figure 4A,C. Gas6 treatment significantly increased ERK activation (1.7-fold; *p* < 0.03; Figure 4B) when compared to controls. Similarly, AKT activation was increased (3.2-fold; *p* < 0.07) by Gas6 in the lungs of treated animals when compared to untreated controls (Figure 4D). The activation of these signaling molecules was decreased to basal levels when the AXL inhibitor was added to the treated animals (Figure 4B,D). Because increased infiltration of cells in the lungs was observed in Gas6 treatment and that previous studies have shown a correlation between ERT and AKT proteins and the modulation of inflammation, we decided to determine levels of secreted lung-associated inflammatory cytokines in this model of PE [20,21,22]. Interleukin 1 alpha (IL-1α) is a pro-inflammatory cytokine that is known to be increased during damage to the epithelium of the lung and has been suggested as a driver of inflammation in disease [23]. IL-1α secretion was significantly increased (1.4-fold; *p* < 003) in the lung of PE animals as compared to controls (Figure 5A). These levels were decreased to basal levels when R428 (AXL inhibitor) was added to the Gas6-treated animals (Figure 5A). Interleukin 2 (IL-2) is a powerful pro-inflammatory cyt okine that promotes the growth and development of peripheral immune cells in an immune response [24,25,26]. It has been suggested to control lung-specific inflammation [26]. Secreted IL-2 levels were significantly increased (1.2-fold; *p* < 0.03) in the lungs of treated animals as compared to controls (Figure 5B). The increased IL-2 was reduced to basal levels with addition of the AXL inhibitor to the Gas6 animals (Figure 5B). Tumor Necrosis Factor alpha (TNFα) is a potent cytokine that can lead to the exacerbation of inflammatory responses with significant roles in many diseases, including pulmonary disorders [27,28]. TNF was significantly increased (1.3-fold; *p* < 0.02) with Gas6 treatment and reduced when the AXL inhibitor was added to these animals (Figure 5C). The coefficient of variation (CV) for the cytokines studied is shown in Figure 5D.

## 4. Discussion

To our knowledge, this is the first report correlating lung signaling to PE driven by Gas6/AXL interaction. The vitamin K-dependent protein Gas6 is a secreted protein expressed in the liver, kidneys, lungs, and placenta [29,30]. This protein has a high affinity for the transmembrane receptor tyrosine kinase (RTK) AXL. Besides the placenta, the AXL receptor is expressed in the lung and is known to be implicated in lung diseases such as cancer [31,32,33]. Gas6/AXL signaling is involved in biological pathways such as cell invasion, metabolism, and immune responses [9,34,35,36]. As previously mentioned, Gas6 is increased in human PE (Gas 6 serum). Interestingly, there are also reports that showed increased AXL during this disease suggesting a role for Gas6/AXL interactions and the development of human PE [37]. PE is a pregnancy-associated hypertensive syndrome known to be associated with a substantial risk for maternal cardiovascular disease (CVD) later in life [34,35,36,38,39]. Previous reports also established high blood pressure as a risk factor of CVD, which suggest that this could be a possible contributor to CVD development in PE patients [40]. As such, the mechanisms of these diseases and disease consequences still need to be evaluated.

We previously reported the development of PE-like symptoms when pregnant rats were treated with Gas6, showing characteristics such as increased proteinuria, and increased inflammation [1]. Knowing that Gas6 and AXL are expressed in the lung and that lung inflammation is suggested as a modifiable risk factor for CVD, we became interested in finding lung responses in this Gas6-induced model of PE [41,42,43]. We first confirmed the development of PE-like characteristics with Gas6 treatment. As previously reported by our lab, we observed increased blood pressure and increased proteinuria in treated pregnant rats as compared to controls. These markers were reduced when the AXL receptor was inhibited, again suggesting a role for PE development in these animals. When examining maternal lungs, we detected lung AXL mRNA and protein levels to be increased in the lungs of the Gas6-treated mothers suggesting lung-derived GAS6/AXL signaling in this model of PE. 

To determine inflammatory profiles, we conducted BALF experiments to initially evaluate to what extent Gas6 treatment contributed to lung inflammation. BALF revealed characteristics previously observed during inflammatory lung conditions. Leukocytic cell quantification revealed increases in total cell quantity and Polymorphonuclear Neutrophils (PMN) abundance in the Gas6-treated animals. Evaluation of total BALF protein abundance was increased during the treatment of pregnant dams. This suggests a possible augmented vascular permeability, which has been associated with airway inflammation. Interestingly we discovered that both total cellular abundance and protein concentration were significantly decreased when the AXL inhibitor was co-administered to mice exposed to Gas6. This suggested a role for GAS6/AXL signaling and the development of lung inflammation in this model of PE. Inflammation modulating molecules are differentially expressed in the lungs in different diseases. Interestingly, rats exposed to Gas6 showed increased levels of the inflammatory cytokines IL-1α, TNF-α, and IL-2. IL-1α is a cytokine involved in both the innate and adaptive immune responses [44,45]. In the lung, IL-1α is associated with inflammation during lung injuries and diseases such as chronic obstructive pulmonary disease (COPD) [46,47]. TNFα is involved in the development of inflammatory diseases such as atherosclerosis, rheumatoid arthritis, and various pulmonary disorders [27]. IL-2 has a novel pro-inflammatory cytokine function for its ability to induce a large panel of trafficking receptor genes for lung inflammation [26]. All of these confirm the infiltration of pro-inflammatory cytokines in the maternal lungs in this model of PE. Furthermore, the fact that the lung infiltration and pro-inflammatory cytokines are reduced during AXL inhibition confirms a possible direct role for Gas6/AXL signaling in lung inflammation and the development of PE. This finding is of importance not only in this model of PE, but also during human PE where levels of Gas6 have been found to be increased in the plasma of the preeclamptic mother [6]. Although studies of lungs during PE are scarce, recent reports have shown that pregnant women infected by the virus COVID-19 (which mostly affects the lungs) have increased lung inflammation and this correlates with obstetric complications such as miscarriages, restricted fetal growth, and most relevant to our studies, the development of PE-like symptoms [48,49,50]. This also correlates with increased serum Gas6 and increased lung AXL in patients affected by this virus [51]. These together suggest, again, a role for lung inflammation and the development of obstetrics complications such as PE. While these experiments clearly show the inducibility of lung inflammation during PE related to Gas6/AXL signaling, this implicates a link between both the lung and the placenta that was previously unknown. We do acknowledge a weakness of this manuscript, in that this was conducted in a PE rodent model of pregnancy rather than a human PE pregnancy. We were aware of this limitation, but our studies originated with findings observed in human studies and modeled in hemochorial placentation, which allowed us to perform studies that could not be otherwise conducted in humans. Follow-up research should focus on aspects of Gas6 and AXL signaling to clarify a possible model to regulate inflammatory responses between lung and placenta. This exploration could provide ways to help ameliorate the effects of PE that require immediate medical intercession.

## Data Availability

All data discussed are presented within the article.

## References

[1] Hirschi K.M., Tsai K.Y.F., Davis T., Clark J.C., Knowlton M.N., Bikman B.T., Reynolds P.R., Arroyo J.A. (2020). Growth arrest-specific protein-6/AXL signaling induces preeclampsia in ratsdagger. Biol. Reprod..

[2] English F.A., Kenny L.C., McCarthy F.P. (2015). Risk factors and effective management of preeclampsia. Integr. Blood Press. Control.

[3] Lecarpentier E., Tsatsaris V., Goffinet F., Cabrol D., Sibai B., Haddad B. (2013). Risk factors of superimposed preeclampsia in women with essential chronic hypertension treated before pregnancy. PLoS ONE.

[4] Barker D.J. (1993). The intrauterine origins of cardiovascular disease. Acta Paediatr..

[5] Challis J.R., Lye S.J., Gibb W., Whittle W., Patel F., Alfaidy N. (2001). Understanding preterm labor. Ann. N. Y. Acad. Sci..

[6] Stepan H., Richter J., Kley K., Kralisch S., Jank A., Schaarschmidt W., Ebert T., Lossner U., Jessnitzer B., Kratzsch J. (2013). Serum levels of growth arrest specific protein 6 are increased in preeclampsia. Regul. Pept..

[7] Laurance S., Lemarie C.A., Blostein M.D. (2012). Growth arrest-specific gene 6 (gas6) and vascular hemostasis. Adv. Nutr..

[8] Korshunov V.A. (2012). Axl-dependent signalling: A clinical update. Clin. Sci..

[9] Tsai K.Y.F., Hirschi Budge K.M., Llavina S., Davis T., Long M., Bennett A., Sitton B., Arroyo J.A., Reynolds P.R. (2019). RAGE and AXL expression following secondhand smoke (SHS) exposure in mice. Exp. Lung Res..

[10] Buurma A.J., Penning M.E., Prins F., Schutte J.M., Bruijn J.A., Wilhelmus S., Rajakumar A., Bloemenkamp K.W., Karumanchi S.A., Baelde H.J. (2013). Preeclampsia is associated with the presence of transcriptionally active placental fragments in the maternal lung. Hypertension.

[11] Aletta B., Marlies P., Frans P., Joke S., Jan Antonie B., Augustine R., Kitty B., Ananth K., Hans B. (2013). OP005. Preeclampsia is associated with the presence of transcriptionally active placental fragments in the maternal lung. Pregnancy Hypertens..

[12] Zieleskiewicz L., Contargyris C., Brun C., Touret M., Vellin A., Antonini F., Muller L., Bretelle F., Martin C., Leone M. (2014). Lung ultrasound predicts interstitial syndrome and hemodynamic profile in parturients with severe preeclampsia. Anesthesiology.

[13] Qu H., Khalil R.A. (2020). Vascular mechanisms and molecular targets in hypertensive pregnancy and preeclampsia. Am. J. Physiol. Heart Circ. Physiol..

[14] Pachtman Shetty S.L., Koenig S., Tenenbaum S., Meirowitz N. (2021). Point-of-care lung ultrasound patterns in late third-trimester gravidas with and without preeclampsia. Am. J. Obstet. Gynecol. MFM.

[15] Diriba K., Awulachew E., Getu E. (2020). The effect of coronavirus infection (SARS-CoV-2, MERS-CoV, and SARS-CoV) during pregnancy and the possibility of vertical maternal-fetal transmission: A systematic review and meta-analysis. Eur. J. Med. Res..

[16] Armaly Z., Jadaon J.E., Jabbour A., Abassi Z.A. (2018). Preeclampsia: Novel Mechanisms and Potential Therapeutic Approaches. Front. Physiol..

[17] Wallace B., Peisl A., Seedorf G., Nowlin T., Kim C., Bosco J., Kenniston J., Keefe D., Abman S.H. (2018). Anti-sFlt-1 Therapy Preserves Lung Alveolar and Vascular Growth in Antenatal Models of Bronchopulmonary Dysplasia. Am. J. Respir. Crit. Care Med..

[18] Reynolds P.R., Mucenski M.L., Le Cras T.D., Nichols W.C., Whitsett J.A. (2004). Midkine is regulated by hypoxia and causes pulmonary vascular remodeling. J. Biol. Chem..

[19] Reynolds P.R., Kasteler S.D., Cosio M.G., Sturrock A., Huecksteadt T., Hoidal J.R. (2008). RAGE: Developmental expression and positive feedback regulation by Egr-1 during cigarette smoke exposure in pulmonary epithelial cells. Am. J. Physiol. Lung Cell. Mol. Physiol..

[20] Hirschi K.M., Chapman S., Hall P., Ostergar A., Winden D.R., Reynolds P.R., Arroyo J.A. (2018). Gas6 protein induces invasion and reduces inflammatory cytokines in oral squamous cell carcinoma. J. Oral Pathol. Med..

[21] Rothlin C.V., Ghosh S., Zuniga E.I., Oldstone M.B., Lemke G. (2007). TAM receptors are pleiotropic inhibitors of the innate immune response. Cell.

[22] Guo G., Gong K., Ali S., Ali N., Shallwani S., Hatanpaa K.J., Pan E., Mickey B., Burma S., Wang D.H. (2017). A TNF-JNK-Axl-ERK signaling axis mediates primary resistance to EGFR inhibition in glioblastoma. Nat. Neurosci..

[23] Borthwick L.A. (2016). The IL-1 cytokine family and its role in inflammation and fibrosis in the lung. Semin. Immunopathol..

[24] Chaplin D.D. (2010). Overview of the immune response. J. Allergy Clin. Immunol..

[25] Liao W., Lin J.X., Leonard W.J. (2011). IL-2 family cytokines: New insights into the complex roles of IL-2 as a broad regulator of T helper cell differentiation. Curr. Opin. Immunol..

[26] Ju S.T., Sharma R., Gaskin F., Fu S.M. (2012). IL-2 controls trafficking receptor gene expression and Th2 response for skin and lung inflammation. Clin. Immunol..

[27] Mukhopadhyay S., Hoidal J.R., Mukherjee T.K. (2006). Role of TNFalpha in pulmonary pathophysiology. Respir. Res..

[28] Lundblad L.K., Thompson-Figueroa J., Leclair T., Sullivan M.J., Poynter M.E., Irvin C.G., Bates J.H. (2005). Tumor necrosis factor-alpha overexpression in lung disease: A single cause behind a complex phenotype. Am. J. Respir. Crit. Care Med..

[29] Wu G., Ma Z., Hu W., Wang D., Gong B., Fan C., Jiang S., Li T., Gao J., Yang Y. (2017). Molecular insights of Gas6/TAM in cancer development and therapy. Cell Death Dis..

[30] van der Meer J.H., van der Poll T., van’ t Veer C. (2014). TAM receptors, Gas6, and protein S: Roles in inflammation and hemostasis. Blood.

[31] Yang D.C., Gu S., Li J.M., Hsu S.W., Chen S.J., Chang W.H., Chen C.H. (2021). Targeting the AXL Receptor in Combating Smoking-related Pulmonary Fibrosis. Am. J. Respir. Cell Mol. Biol..

[32] Boshuizen J., Pencheva N., Krijgsman O., Altimari D.D., Castro P.G., de Bruijn B., Ligtenberg M.A., Gresnigt-Van den Heuvel E., Vredevoogd D.W., Song J.Y. (2021). Cooperative Targeting of Immunotherapy-Resistant Melanoma and Lung Cancer by an AXL-Targeting Antibody-Drug Conjugate and Immune Checkpoint Blockade. Cancer Res..

[33] Fujino N., Kubo H., Maciewicz R.A. (2017). Phenotypic screening identifies Axl kinase as a negative regulator of an alveolar epithelial cell phenotype. Lab. Investig..

[34] Rana S., Lemoine E., Granger J.P., Karumanchi S.A. (2019). Preeclampsia: Pathophysiology, Challenges, and Perspectives. Circ. Res..

[35] Osol G., Bernstein I. (2014). Preeclampsia and maternal cardiovascular disease: Consequence or predisposition?. J. Vasc. Res..

[36] Bokslag A., van Weissenbruch M., Mol B.W., de Groot C.J. (2016). Preeclampsia; short and long-term consequences for mother and neonate. Early Hum. Dev..

[37] Ozakpinar O.B., Sahin S., Verimli N., Simsek G.G., Maurer A.M., Eroglu M., Tetik S., Uras F. (2016). Association between the growth arrest-specific 6 (Gas6) gene polymorphism c.834 + 7G>A and preeclampsia. J. Matern. Fetal Neonatal Med..

[38] Popescu P., Nanea T. (1986). Thoracic pain syndrome appearing after acute myocardial infarction. Rev. Med. Interna Neurol. Psihiatr. Neurochir. Dermatovenerol. Med. Interna.

[39] Wu P., Haththotuwa R., Kwok C.S., Babu A., Kotronias R.A., Rushton C., Zaman A., Fryer A.A., Kadam U., Chew-Graham C.A. (2017). Preeclampsia and Future Cardiovascular Health: A Systematic Review and Meta-Analysis. Circ. Cardiovasc. Qual. Outcomes.

[40] Fuchs F.D., Whelton P.K. (2020). High Blood Pressure and Cardiovascular Disease. Hypertension.

[41] Van Eeden S., Leipsic J., Paul Man S.F., Sin D.D. (2012). The relationship between lung inflammation and cardiovascular disease. Am. J. Respir. Crit. Care Med..

[42] King P.T. (2015). Inflammation in chronic obstructive pulmonary disease and its role in cardiovascular disease and lung cancer. Clin. Transl. Med..

[43] Fujimori T., Grabiec A.M., Kaur M., Bell T.J., Fujino N., Cook P.C., Svedberg F.R., MacDonald A.S., Maciewicz R.A., Singh D. (2015). The Axl receptor tyrosine kinase is a discriminator of macrophage function in the inflamed lung. Mucosal Immunol..

[44] Cavalli G., Colafrancesco S., Emmi G., Imazio M., Lopalco G., Maggio M.C., Sota J., Dinarello C.A. (2021). Interleukin 1alpha: A comprehensive review on the role of IL-1alpha in the pathogenesis and treatment of autoimmune and inflammatory diseases. Autoimmun. Rev..

[45] Malik A., Kanneganti T.D. (2018). Function and regulation of IL-1alpha in inflammatory diseases and cancer. Immunol. Rev..

[46] Rabolli V., Badissi A.A., Devosse R., Uwambayinema F., Yakoub Y., Palmai-Pallag M., Lebrun A., De Gussem V., Couillin I., Ryffel B. (2014). The alarmin IL-1alpha is a master cytokine in acute lung inflammation induced by silica micro- and nanoparticles. Part. Fibre Toxicol..

[47] Terlizzi M., Colarusso C., Popolo A., Pinto A., Sorrentino R. (2016). IL-1alpha and IL-1beta-producing macrophages populate lung tumor lesions in mice. Oncotarget.

[48] Mendoza M., Garcia-Ruiz I., Maiz N., Rodo C., Garcia-Manau P., Serrano B., Lopez-Martinez R.M., Balcells J., Fernandez-Hidalgo N., Carreras E. (2020). Pre-eclampsia-like syndrome induced by severe COVID-19: A prospective observational study. BJOG.

[49] Rebutini P.Z., Zanchettin A.C., Stonoga E.T.S., Pra D.M.M., de Oliveira A.L.P., Deziderio F.D.S., Fonseca A.S., Dagostini J.C.H., Hlatchuk E.C., Furuie I.N. (2021). Association Between COVID-19 Pregnant Women Symptoms Severity and Placental Morphologic Features. Front. Immunol..

[50] Rad H.S., Rohl J., Stylianou N., Allenby M.C., Bazaz S.R., Warkiani M.E., Guimaraes F.S.F., Clifton V.L., Kulasinghe A. (2021). The Effects of COVID-19 on the Placenta During Pregnancy. Front. Immunol..

[51] Morales A., Rojo Rello S., Cristobal H., Fiz-Lopez A., Arribas E., Mari M., Tutusaus A., de la Cal-Sabater P., Nicolaes G.A.F., Ortiz-Perez J.T. (2021). Growth Arrest-Specific Factor 6 (GAS6) Is Increased in COVID-19 Patients and Predicts Clinical Outcome. Biomedicines.

